# Theorizing community health governance for strengthening primary healthcare in LMICs

**DOI:** 10.1093/heapol/czac002

**Published:** 2022-01-25

**Authors:** Shirin Madon, S Krishna

**Affiliations:** Department of International Development/Department of Management, London School of Economics & Political Science, Houghton Street, London WC2A 2AE, UK; Foundation for Research in Health Systems and Indian Institute of Management, Bangalore, India

**Keywords:** Community health governance, primary healthcare, VHSNCs, India

## Abstract

In recent years, community health governance structures have been established in many low and middle-income countries (LMICs) as part of decentralization policies aimed at strengthening primary healthcare systems. So far, most studies on these local structures either focus on measuring their impact on health outcome or on identifying the factors that affect their performance. In this paper we offer an alternative contribution that draws on a sociological interpretation of community health governance to improve understanding of how the government’s policy vision and instrumentation translate to interactions that take place within local spaces at field level. We study 13 Village Health Sanitation and Nutrition Committees (VHSNCs) in Karnataka, India, from 2016 to 2018 focusing on sanitation, nutrition and hygiene which remain impediments to improving primary healthcare amongst poor and marginalized communities. Three local governance mechanisms of horizontal coordination, demand for accountability and self-help help to explain improvements that have taken place at village level and contribute to the creation of a new theory of community health governance as evolving phenomenon that requires a constant process of learning from the field to strengthen policymaking.

**Key messages**

- This paper draws on a sociological interpretation of community health governance as evolving phenomena from policy to practice.
- We adopt a processual approach to analyse the links between policy intent, instrumentation and interaction at field level.
- Policymakers need to assimilate the learnings from community health governance structures in order to improve primary healthcare.

## Introduction

Decentralization has been a major strategy for improving primary healthcare systems in LMICs with much effort directed at identifying optimal structures for granting ‘decision space’ to lower-level bureaucrats to address key aspects of governance such as resource allocation and performance measurement (Bossert, 1998; Roman *et al.*, 2017). In recent years, community health governance has emerged as a key policy mechanism for decentralizing primary healthcare through the establishment of ‘invited spaces’ where local functionaries, politicians and civil society representatives are encouraged to participate in improving primary healthcare (McCoy *et al.*, 2012; Abimbola *et al.*, 2016). Village committees established by health ministries in India, Tanzania, Bangladesh and South Africa to improve primary healthcare through community participation and capacity-building constitute one type of invited space (Cornwall, 2002; Aiyar, 2010). So far, much of the current discourse on community health governance focuses on the operational aspects of these invited spaces, e.g. whether village health committees are established according to government guidelines and key contextual challenges faced in doing so (George *et al.*, 2015; Madon *et al.*, 2018).

**Figure 1.**
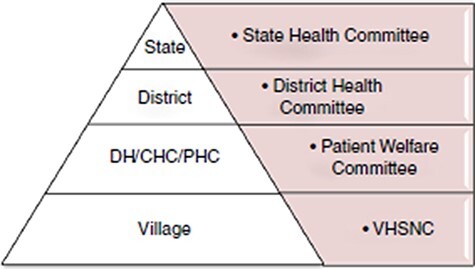
The VHSNC in relation to higher levels of the health planning apparatus in India.

Source: Government of India (2015)

In this paper we offer an alternative sociological approach for the study of community health governance by drawing on the concept of interactive governance first proposed by Kooiman *et al.* (2005) based on their empirical research in the fisheries sector in LMICs. We propose that this theoretical lens can help to provide a process-oriented insight into how community health governance unfolds from policy to practice. In particular, while ‘participation’ and ‘capacity-building’ are key hallmarks of community health governance strategies by governments, we seek to characterize how these are enacted at the level of practice by studying Village Health Sanitation and Nutrition Committees (VHSNCs) established in 2008 by India’s National Health Mission (NHM)^1^. Figure 1 describes the position of the VHSNC in relation to higher levels of the health apparatus in India. Although established over a decade ago, the country continues to lag behind many other developing countries in terms of improving sanitation, nutrition and hygiene, with water-borne diseases such as diarrhoea and cholera still major killers and low birth weight among the highest in the world (Chakraborty, 2017). Many VHSNCs in India remain largely non-functional with much interest among scholars to capture the perspectives of key functionaries regarding the implementation challenges that prevent improvements in primary healthcare (Scott *et al.*, 2017; Ved *et al.*, 2018; Dhiman *et al.*, 2020; Abdul Azeez *et al.,* 2021). While these studies provide insights into the challenges faced by VHSNCs at the level of implementation, they fall short of generating an understanding of how these invited spaces are used and how that usage changes over time. An exception to this is a longitudinal study of VHSNCs in Karnataka which the authors undertook to trace the frequency and quality of interactions among committee members as they developed a set of practices to make them accountable to each other (Madon and Krishna 2017). We provide more details of this earlier study in the methodology section as it forms the backdrop for the current study in which our focus is on learning how VHSNCs that have already achieved a basic level of functioning develop enhanced capacity to improve sanitation, nutrition and hygiene.

In the next section we develop our theoretical framework for studying community health governance which is followed by a description of our research methodology. We then present our case study of VHSNCs in Gumballi Primary Health Centre (PHC), Karnataka, from 2016 to 2018. Finally, we draw implications from our study to sensitize policymakers engaged in community health governance initiatives as part of decentralized primary healthcare agenda.

## Theorising community health governance as interaction

Invited spaces in the form of local committees have been established in many LMICs as part of policy to strengthen community participation and capacity-building for a range of essential services such as healthcare. These spaces have a semi-autonomous existence as they are partially embedded within the institutional apparatus of the state and therefore shaped by its principles, guidelines and structures yet allow community members to take decisions about prioritizing local action and spending (Cornwall, 2004). In the literature, invited spaces are conceptualized as both ‘the basis for action and the field of action’ (Lefebvre, 1991; Visser *et al.*, 2021). As the basis for action, an invited space such as a village health committee embodies a particular vision or intent held by the government about its value for promoting service delivery. For example, such a policy may be based on the belief that it will help mobilize the community to participate in local planning and monitoring, self-organize, take decisions and hold frontline agents to account to improve primary healthcare (Madon and Krishna 2017). Based on its vision, the government will put in place procedures and guidelines to establish locally demarcated meeting spaces for community participation to occur (Aiyar, 2010). For example, Visser *et al.* (2021) observe how four invited spaces were envisioned and structured by the Dutch government to encourage citizens to develop capacity for self-organization and collective action to improve service delivery.

While invited spaces may provide the ‘basis for action’, they only become a ‘field of action’ through the reflexive capabilities of individual actors as they undertake repetitive action in arenas where people interact such as in a village committee. A noticeable gap in the community health governance literature relates to understanding the interplay between these two facets of invited spaces. In this paper, we aim to address this by drawing on the concept of interactive governance from the social sciences which provides a useful lens to improve understanding of the link between policy and practice in key societal areas such as poverty alleviation and public health (Kooiman *et al.*, 2005). The central element in Kooiman’s governance approach is that of interaction which refers to the day-to-day activities that are undertaken to address challenges of service delivery and improve a situation, e.g. within an invited space such as a village health committee. As Kooiman *et al.* (2008) note, interactions at field level can also take unexpected forms and lead to unanticipated outcomes as a result of the dynamics that occur between actors working together to address societal challenges. Of interest is to understand the connection between the intent of government policy, the structures or institutional arrangements that governments put in place to achieve the intent, and how these are interpreted and enacted at village level.

**Figure 2.**
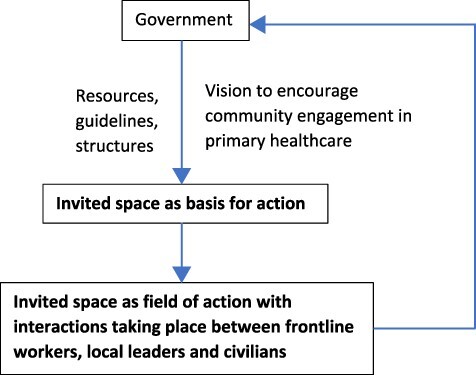
Theorizing community health governance as interaction

This paper contributes to the discourse on community health governance in LMICs by drawing on the perspective of interactive governance to explore transitions in terms of intent, instrumentation and interactions that take place within invited spaces. We investigate the formation of VHSNCs both as the basis for action (in terms of intention and instrumentation of government) and as a field of action (in terms of interaction between VHSNC members). Our focus is on activities related to sanitation, nutrition and hygiene recognized as key social determinants of health for reducing the incidence of water-borne diseases and thereby improving primary healthcare. By recognizing community health governance as evolving phenomena rather than as a static policy intervention, our contribution is aimed at building theory about the temporal progression of activities that take place at field level as elements of explanation and understanding for improving primary healthcare as illustrated in Figure 2.

## Research methodology

We adopt a qualitative, longitudinal case study approach recognized as appropriate for conducting process research where the focus is on studying how phenomena unfold over time (Langley *et al.*, 2013). Our case is of the enactment of policy processes related to sanitation, nutrition and hygiene in 13 VHSNCs of Gumballi PHC, Karnataka, which were identified as well-functioning by the authors in an earlier study conducted from 2012 to 2015 (Madon and Krishna 2017). In that study, the authors had traced the different stages of maturity through which the VHSNCs transitioned from being non-functional in 2012 to achieving a basic level of functioning in 2015. The authors described how these interactions included sensitizing committee members about their roles and responsibilities during their enrolment onto the committee and how this led to a set of practices that made them accountable to each other for improving village health.

Our rationale for selecting the same set of VHSNCs is based on evidence accumulated from previous studies that demonstrates how intersectoral collaboration requires the capacity for sustaining a certain level of community social relations and negotiations between diverse committee members (Kim *et al.*, 2017). Gumballi PHC located in the Yelandur administrative region of Chamarajanagar district of Karnataka, 176 km away from the state capital, Bangalore, serves 13 villages. This cluster of villages has a total population of around 1800 as per the last census in 2011 consisting of mainly semi-arid, rain-dependent flatlands along with forested hills. With a literacy rate of 58%, the district is one of the less-developed areas of Karnataka and since 2000 has suffered from severe drought conditions. While there are differences between the villages in terms of size or caste composition, for the purpose of this study we focus more on their similarities in terms of community health governance processes that take place within the VHSNCs.

### Data collection and analysis

We collected data for this study through documentary sources and participant observation. Documentary sources published by the NHM programme and other government bodies were used to understand how the government first envisioned the VHSNC as a forum for promoting intersectoral collaboration and the instruments it has used for this purpose. Participant observation was used to study the VHSNCs as a field of action focusing on the interactions that took place between committee members within the village setting. Observational data were collected by two locally-based and experienced Kannada-speaking^2^ researchers who had already built a good relationship with VHSNC committee members in our study villages through earlier fieldwork from the time the committees were first established to their transition to a functional status (Madon and Krishna 2017). When we commenced this research in 2016, the 13 VHSNCs were serving as regular forums for health workers to impart awareness to members about the importance of sanitation, nutrition and hygiene for maintaining good health (Madon and Krishna 2017). The field researchers attended a total of 375 meetings over the 3-year period from 2016 to 2018 in order to study the nature of interactions that were taking place within the VHSNCs related to these three key social determinants. During this period no Gram Panchayat elections were held in Gumballi which could potentially have influenced the behaviour of members, particularly of local political representatives.

VHSNC meetings usually took place every month and lasted approximately 45 minutes. Free-form notes were taken by our fieldworkers at each meeting which were then transcribed and discussed soon after each field trip with one of the authors whose mother tongue is Kannada in order to guard against selectivity in the findings. Once this exercise was completed, the field observations were translated into English and sent across to the London-based researcher who logged the data in a coding template that contained a village-wise record of each meeting, attendance registers, what was discussed and by whom, disagreements that arose, and action points. During the 3-year period, triangulation between the research team members was undertaken through a mix of online and face-to-face meetings.

In terms of analysis, we used our observational data from the VHSNC meetings to create an annual activity occurrence index for each village based on the number of occurrences of action that took place related to the three intersectoral activities of sanitation, nutrition and hygiene. Plotting activity data against time provided us with a visual representation of the increased incidence of intersectoral activities and led to the main part of our analysis of patterns or themes in the interactions that were taking place at VHSNC level. The field-level data were then coded according to three key governance constructs observed during committee meetings. We adopted a within-case approach to enable the many details present in the narrative to emerge and be interpreted by the reader guided by the study’s theoretical framing (Eisenhardt and Graebner 2007).

Our study did not involve human participants or data relating to identifiable human subjects and we explained the objectives of our research to VHSNC members having obtained prior approval for the conduct of this research from Gumballi PHC.

## Results

### Intersectoral collaboration in 13 VHSNCs of Gumballi PHC, Karnataka, from 2016 to 2018

#### Government vision and instrumentation

The VHSNCs were established in 2008 by the NHM in order to improve primary healthcare through community engagement. We studied five documents that were published by the NHM over time to understand the government’s vision behind this policy intervention and its instrumentation strategy as detailed in Table 1. The documents published in 2014 are the latest publicly available sources.

**Table 1.** Government of India vision and instrumentation of VHSNCs from five sources

1 Meeting people’s health needs in rural areas: framework for implementation 2005–2012, NRHM	2 Guidelines regarding constitution of VHSNCs and utilization of untied grants to these committees, 2010	3 Framework for implementation 2012–2017, NHM	4 Guidelines for community processes, NRHM, 2014	5 Handbook for members of VHSNC, NHM, 2015
VHSNC^a^ to empower community to take leadership in health matters	VHSNC to be structured under umbrella of Gram Panchayat	VHSNC to be structured under umbrella of Gram Panchayat	VHSNC as platform for community to be informed about and participate in health programmes	
Gram Panchayat to take lead in management of village health	Use of untied grant for eligible expenses	VHSNC to monitor service delivery of health functionaries	Platform for community to articulate needs and for action to be taken to address social determinants of health	
Adequate representation of disadvantaged sections of the community	Adequate representation of disadvantaged and NGOs	VHSNC to support convergence of social determinants	VHSNC under umbrella structure of Gram Panchayat which should be empowered to take a leadership role in village health governance and promotion of collective action	
	VHSNC to be accountable for expenses, survey data and records	VHSNC training to be given	Listen to the voice of lay service users, especially mothers	Health as a basic human right for which government is responsible to provide
	VHSNCs to be given orientation and training after establishment		Measure VHSNC performance based on meeting frequency, untied grant spent on eligible activities, good record-keeping, achievement of children frequenting anganwadi^b^ centre, numbers of institutional delivery, number of non-functional hand pumps in village, households with toilets, school midday meal and use of mosquito nets by pregnant women	

a The VHSNCs were earlier known as VHSCs. In 2011, an order was issued to expand the committee’s role to include nutrition.

b An anganwadi centre is a rural childcare centre in India.

From the documents reviewed, we identified three aspects related to government vision and instrumentation. First, the government has been explicit from the start in portraying an image of the VHSNC as an institution established to ‘empower’ the community to take decisions related to improving village health with key instruments being the annual untied grant of Rs. 10 000 (approx. US$152) and the guidelines for eligible expenses. In Documents 1–3, mention of the Gram Panchayat^3^ was in terms of how it provided an overall governance structure within which the VHSNC was one of its subcommittees specifying operating procedures for electing members. In the later Documents 4 and 5, the focus shifted away from the Gram Panchayat’s primarily structural role towards an image of the Gram Panchayat itself becoming empowered through the VHSNC and taking on ‘a leadership role in village health governance and collective action’. This shifting narrative of the Gram Panchayat’s role in village health receives support from other wider policy directives during this period. For example, in 2015 the 14th Finance Commission stressed a three-fold rise in the funding of rural local government in India with the Gram Panchayats receiving the largest share of finances (Salve and Tewari 2015).

Second, the government’s vision for establishing the VHSNCs reflects a focus on including different sections of the community in village health governance. Documents 1 and 2 were explicit in providing guidelines for the recommended percentage of VHSNC members from different socio-economic backgrounds and marginalized groups such as women and minorities. By the time Document 4 was published there was an explicit mention of the need to ‘listen to the voices of lay service users, especially mothers’. However, while all the documents reviewed acknowledge the need for the VHSNCs to institutionalize community participation, they do not specify how this can occur. This observation concurs with the challenges observed in relation to village-level water governance structures in Karnataka where operational and management functions were successfully devolved to the Panchayati Raj institutions at the block level but failed to motivate household participation (Raghavan *et al.*, 2017).

Third, in the earlier sources, Document 3 mentioned the intent of VHSNCs to ensure convergent action on the social determinants of health. In subsequent Documents 4 and 5, the government increasingly presents the VHSNC as an intersectoral body by providing a detailed checklist to explain each social determinant, its value in overall health promotion and a detailed list of performance criteria for measuring sanitation, nutrition and hygiene improvements. However, the documents reviewed were silent about the intersectoral support required from higher levels of government. For example, two departments are involved in the implementation of essential maternal and child nutrition interventions, the Department of Health and Family Welfare and Women and Child Development with protocols for collaboration identified at strategic level. However, as corroborated by other publications, at an operational level, sectoral domination of the health department has resulted in over-emphasis on improving infant and maternal mortality indicators rather than on the implementation of relevant nutritional interventions aimed at prevention (Kim *et al.*, 2017).

*Observations related to the frequency of VHSNC meetings, attendance of members and regularity of actions taken to improving primary healthcare.*

Table 2 provides details of meeting frequency and attendance in the 13 villages across the study period. In the majority of villages there were between 8 and 11 meetings held each year apart from the village of Gangavadi where the committee lacked an active Accredited Social Health Activist (ASHA) worker to convene meetings until 2018 and BR Hills which was a latecomer in terms of establishing a VHSNC. A representative group of between 11 and 14 community members has participated in the VHSNC meetings across the three years with participation of marginalized^4^ caste groups and women representatives remaining stable over the duration of our research.

**Table 2.** Meeting frequency and attendance at VHSNCs in Gumballi from 2016 to 2018

	2016			2017			2018		
	No. of meets	Avg. members per meet	% marginalised	No. of meets	Avg. members per meet	% marginalised	No. of meets	Avg. members per meet	% marginalised
BR Hills	3	14	25	9	13	18	10	14	20
Changachalli	6	14	33	6	14	33	8	12	23
Dasanahundi	12	12	32	10	11	21	9	11	20
Gangavadi	2	12	29	0	0	0	6	12	17
Ganiganur	12	13	28	11	12	20	11	12	20
Gumballi	11	15	26	12	12	25	10	11	24
Hegadehundi	11	11	25	7	11	28	10	11	24
Komanapura	12	12	24	10	11	26	10	12	25
Krishnapura	10	11	30	8	13	28	8	13	24
Uppinamole	11	11	32	8	11	32	10	11	23
Vadagere	10	13	30	9	12	32	8	12	24
YK Mole	11	15	30	12	14	33	11	14	25
Yargamballi	11	15	28	10	13	32	11	12	25

**Figure 3.**
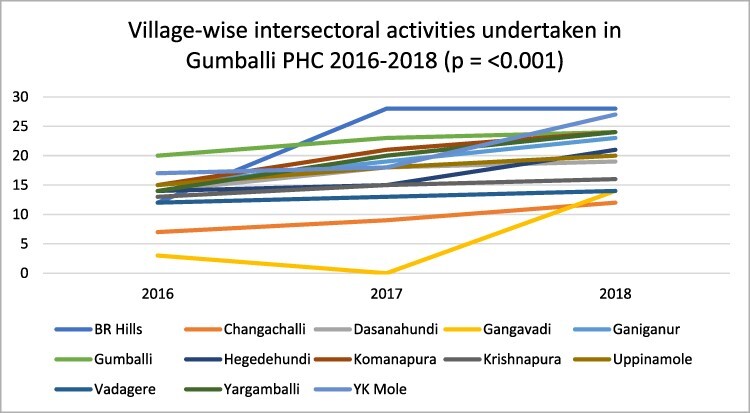
Illustrating the trend in village-wise intersectoral activities between 2016 and 2018

Overall, the data suggest that there has been a consistent commitment in Gumballi’s VHSNCs to meet and engage in discussions about improving village health. Plotting the number of activities that occurred related to key social determinants of sanitation, nutrition and hygiene in each of the 13 villages over the 3-year period revealed an increasing trend as illustrated in Figure 3.

#### The forms of actions that have taken place in Gumballi’s VHSNCs

We identify three key governance structures to characterize the type of action we observed: horizontal coordination; demanding accountability and self-help and show how they have been mutually reinforcing.

#### Horizontal coordination

From 2016, we observed that coordinated action between health workers was common place with the auxiliary nurse midwide (ANM) asking the ASHA and anganwadi workers^5^ to weigh children to find out if they were malnourished and to make parents aware of how to prevent and treat the condition. In the majority of VHSNCs, the anganwadi worker took the lead in discussions about the prevalence and extra supplements for low-birth-weight children in the village. We observed how this team of three frontline workers was particularly active during the summer and monsoon months informing members during house-to-house visits taking place to identify 0–5-year children for oral rehydration salts (ORSs) and zinc tablets to curb the onset of loose motions and diarrhoea. In over half of our study VHSNCs, it was due to the instigation of several members of the VHSNC including the president and school teacher that anganwadi centres started demonstrations to show community members how to administer ORS nutritional supplements.

In the majority of the VHSNCs, activities related to hygiene and cleanliness were initiated by non-health workers including lay persons. The VHSNC president was observed to coordinate works that affected the whole village such as blocked drains or preparing for a religious festival but was also increasingly proactive about matters related to personal hygiene and cleanliness which had earlier been the domain of ASHA workers, e.g. by asking the ASHA worker to impart awareness about menstrual hygiene to women and girls to avoid fungal infections.

The school teacher was equally vocal in matters related to hygiene and cleanliness around the vicinity of the school, urging parents to ensure household hygiene for preventing worm infections among children. Similarly, the village waterman who would routinely check the status of water sources around the village began to proactively notice garbage lying around the village which he reported to the VHSNC. The vignette below provides an illustration of horizontal coordination that took place around issues related to hygiene and cleanliness.

**Table:** 

In a 2016 meeting held in Dasanhundi village, the VHSNC president and committee members were involved in a discussion about village cleanliness. The school teacher raised the issue of lack of cleanliness in the school and that children and villagers were throwing paper and other waste items in front of the school which resulted in rubbish coming into the school compound and the need for a dustbin to be placed inside the premises. A general member also raised a concern that the public are throwing waste items in drains and in the roads which eventually causes drains to become blocked and requested the committee to purchase and place a cement dustbin near the temple and school area. The president gave assurances that dustbins would be placed in these two different places in the village and informed the secretary to obtain a quotation for the items. The following month, the committee approved the purchase of the two dustbins from the VHSNC fund which were placed in the two locations.

In 2017, horizontal coordination amongst VHSNC members was apparent in the implementation of two new government programmes. The Mathru Poorna Yojana scheme run by the Women and Child Development Department was launched to provide a free nutritional meal to pregnant women and lactating mothers in the anganwadi centre. However, the anganwadi workers reported that many women who were eligible for the free meal were not visiting the centre and preferred instead to take the food rations home because it was not customary for a woman to leave her home unaccompanied. The VHSNC president had so far not been aware of this situation and the committee provided the space for an open discussion of this issue and its possible resolution in the presence of pregnant and lactating mothers. A second programme launched that year by the Health and Family Welfare Department was the Intensified Diarrhoea Control Fortnight programme. In the majority of our study VHSNCs, together with health workers the VHSNC president and school teacher were actively engaged in disseminating messages to members and the wider community particularly about the treatment of diarrhoea in very-low-birth-weight and severely malnourished children.

By 2018, we observed how the Gram Panchayat began to take responsibility for hygiene and cleanliness in the village. For example, in a few villages the panchayat initiated the building of a fence around the anganwadi centre to stop people throwing garbage and urinating, issuing a formal complaint letter to households who violated this. In other cases, the president suggested conducting regular jathas (street plays) to sensitize villagers about cleanliness.

#### Demand for accountability

Over the 3-year period of our study, there has been an increasing demand for accountability from VHSNC members including lay persons. Questions have been directed at health workers and the waterman about the results of tests undertaken to check the quality of village water sources and the purpose of the house-to-house larva surveys. We particularly noticed that lay persons were becoming more vocal in highlighting the Gram Panchayat’s responsibility for village health as illustrated by the complaint letter written by Dasanhundi VHSNC members.

**Table:** 

Complaint letter about water tank cleaning in Dasanhundi village In the June 2018 VHSNC meeting held in Dasanhundi village, two general members raised the urgent need for the panchayat to take responsibility to clean the drinking water tanks which had turned green due to ants and insects entering because the water tank was not properly covered. The VHSNC president replied that he had informed the panchayat 2–3 times but the secretary and others were not doing the work. Eventually, the Dasanhundi VHSNC members decided to write a letter to the panchayat president. The letter was written during the meeting and signed by all the members present. Once the letter was presented to the panchayat, the issue was discussed in the July panchayat meeting which was agreed in August after which all the village water tanks were subsequently cleaned.

The VHSNC meetings have increasingly provided a forum for the president and other members of the committee including mothers to become increasingly vocal in complaining about the quality of food served in the anganwadi centres as illustrated below.

**Table:** 

Complaint about quality of nutritional food in Yargamballi village In a 2018 meeting held in Yargamballi village, the ASHA raised the issue of supplementary nutritional services (SNP) available for pregnant ladies and lactating mothers who were complaining that over the past six months in two of the anganwadi centres in the village there have been irregularities in the timing and amount of SNP distribution. During the meeting, when the president asked the concerned anganwadi workers about this complaint they did not accept the allegation. When the committee members decided to write a letter to the anganwadi supervisor, the anganwadi workers agreed about the irregularities of SNP distribution to these beneficiaries. The president emphasized the importance of VHSNC members regularly checking the functioning of the anganwadi centres for SNP distribution to pregnant ladies and lactating mothers and for providing food to children. The president said he will inform all panchayat members in a future panchayath meeting to check the quality of distribution at the anganwadi centre. At the next VHSNC meeting, the ASHA worker informed the committee that SNP distribution had been given at the right time and quality according to guidelines issued by the Women and Child Development Department.

#### Self-help

Over our study period, messages from health workers and non-health VHSNC members were increasingly aimed at encouraging individual households to help themselves. For example, the president and waterman targeted individual households as being responsible for reviewing their water storage practices to ensure that containers were cleaned every 2–3 days. In the majority of the VHSNCs, the president and waterman actively encouraged members to regard village water sources as a public good by reporting damaged water pipes and unclean water tanks and closing water taps when not in use to avoid stagnant water accumulating in drains where mosquitos could breed. Similarly, a strong message was communicated by the president and health workers about personal and household hygiene, e.g. the need for individuals to help themselves by wearing long-sleeved clothes, using insecticide materials in the home and ensuring cleanliness in the kitchen.

The increasing acceptance of self-help as a solution for improving village health manifested itself by members requesting the results of the water sample taken from the village to the PHC lab for testing, as well as the results of water samples taken from storage containers during house-to-house larva surveys. A self-help form of action also manifested itself at the village level as the VHSNC president was observed to demonstrate a growing sense of responsibility, e.g. by undertaking an exercise of counting how many village taps needed to be repaired and to follow through by ensuring that the repair actually took place either through VHSNC funds, or with support from the Gram Panchayat.

From 2016, there was an increasing onus on parents to put in place basic hygiene practices in their home to prevent and treat worm infections amongst children. Similarly, the messaging of self-help from VHSNC members was important for the implementation of the Intensified Diarrhoea Control fortnight programme as the VHSNC president and school teacher encouraged households to take responsibility for treating diarrhoea by prepositioning ORS supplements in the household in case of need, rather than relying on health workers. The focus on household responsibility became stronger from 2017 when an important topic initiated by the president during VHSNC meetings related to the need for community members to construct individual toilets in the household. Gram Panchayat members, health workers, school teacher, waterman and anganwadi workers together encouraged particularly pregnant women to replace open defecation practices with toilet construction using government funds.

Overall, the clear message communicated by the VHSNC president was that sanitation, nutrition and hygiene was the responsibility of everyone, not just the government. This overriding message was reinforced through the central government’s Clean India, Health India campaign where emphasis was placed on households to lift themselves out of the cycle of ill-health as a matter of pride for themselves and their village.

#### Within-case analysis

We draw on our field-level observations of Gumballi’s VHSNCs to improve understanding of community health governance. The government’s vision in establishing the VHSNCs as a basis for action dedicated to improving primary healthcare contained three key elements. The first was to promote devolution and community engagement in primary healthcare through policy support and funding to the Gram Panchayat. The second was to promote an inclusive form of community engagement at village level. In doing so, the government issued guidelines for VHSNC membership which initially included marginalized community representatives and was later extended to include lay persons, with specific mention of pregnant women and lactating mothers. The third element was the urgent need to focus on improving sanitation, nutrition and hygiene as key determinants of village health with training manuals and guidelines detailing key indicators for measuring each determinant.

The government’s policy vision, intent and instrumentation strategy have been important enablers for establishing the VHSNC as the basis for improving primary healthcare at village level. However, this is a necessary but insufficient condition for actual improvements to be achieved in primary healthcare as the latter depends crucially on how the VHSNCs move from being a basis for action to a field of action. Our findings of VHSNC functioning in Gumballi provide some insights into village-level mechanisms that have helped to translate ‘vision’ into ‘action’ through the reflective capabilities of individual actors as they undertake repetitive action to improve village health. The government’s vision for devolution in primary healthcare finds expression at the field of action through the emergence of a mutually reinforcing relationship between the VHSNC and the Gram Panchayat. On the one hand, the regularity of interactions has made it common place for the VHSNC to request both political support and funding from the Gram Panchayat for essential works needed to improve village health. On the other hand, the Gram Panchayat through its engagement with the VHSNC has enhanced its position as a focal village development body. As the relationship between the two village bodies has evolved, so too has the way in which accountability is manifested.

Our findings show that three key governance constructs of horizontal coordination, demand for accountability and self-help influence each other and have combined to move the VHSNCs from being a basis for action to a field of action. The increased pace of interactions between VHSNC members and lay persons has resulted in greater demands for accountability with respect to improving different aspects of sanitation, nutrition and hygiene. At the same time, we see a new localized accountability environment emerging, which can be described under the label of ‘self-help’ manifesting itself at different levels as individuals and households have started to take responsibility for sanitation, nutrition and hygiene. At a higher level, and despite the social hierarchies that exist within any village environment, the VHSNC and Gram Panchayat have developed a shared understanding for self-help as a strategy for improving village health. For example, as it assumes an increasing leadership role in issues related to village health, the Gram Panchayat undertakes responsibility for diagnosing problems and taking action to ensure their eventual resolution such as undertaking the routine surveillance of village waterworks and ensuring that repairs have been carried out.

The regularity of interactions taking place within the VHSNCs has resulted in greater opportunities for coordination in the efforts to improve sanitation, nutrition and hygiene in the villages. We identified cases of how members have engaged in tasks which do not necessarily correspond to their formal line of duty, e.g. with the waterman routinely involving himself in issues related to garbage collection. Similarly, the inclusion of lay persons in the VHSNC while mandated as a guideline issued by the government has over time found expression at the field of action as these community members have become increasingly involved in monitoring and reporting problems related to lack of sanitation, nutrition and hygiene. Through open discussion during the VHSNC meeting and despite their eligibility for availing of a nutritional meal, lay persons such as pregnant women and lactating mothers disclosed the social and cultural reasons for their reluctance to obtain a nutritious meal at the anganwadi centre.

**Figure 4.**
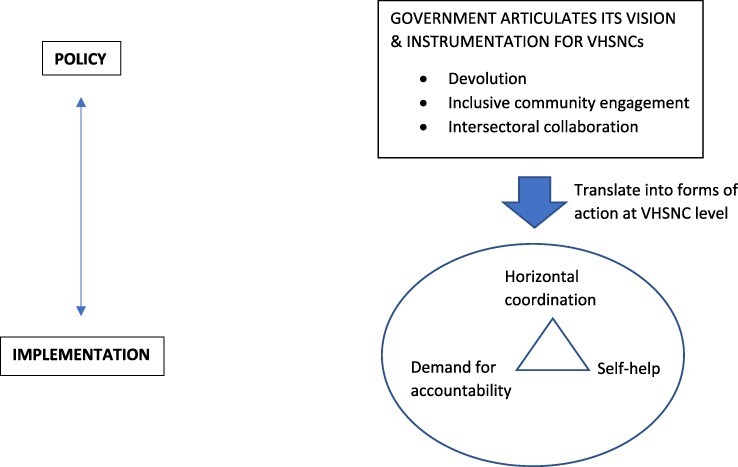
Gumballi’s VHSNCs as basis for action and form of action

Figure 4 summarizes the mechanisms through which government intent and instrumentation with regard to its establishment of the VHSNCs translate to tangible outcome at village level. The three local governance mechanisms of horizontal coordination, demand for accountability and self-help identified through our empirical study in Gumballi help to explain the improvements that have taken place in primary healthcare and in doing so provide an opportunity to contribute to the creation of a new theory of community health governance to which we now turn.

## Discussion and conclusion

Our aim in this paper has been to theorize community health governance activity drawing on the concepts of invited spaces and interactive governance. This framing enabled us to study the experience of VHSNCs in Gumballi as evolving phenomena from their establishment by government as the basis for action to the interactions that have taken place between committee members at village level at the field of action. As stated earlier, the context for our study was the catchment of 13 VHSNCs in Gumballi PHC which had been identified as well-functioning by the time we commenced our research in 2016 (Madon and Krishna 2017).

Our study offers a new theory of effective community health governance that helps to explain how policy aimed at decentralizing primary healthcare interacts with implementation. In particular, our theorization has helped to shed light on what has worked with the policy process and why. To test our theory, we compare our results with the critical factors that have been identified in other recent studies of VHSNCs as proxies for success or failure. In order to do so, we have selected five representative studies published between 2016 and 2021 that aimed to evaluate the VHSNCs in different parts of India as described in Table 3. The studies were mainly qualitative and cross-sectional. In these studies, macro-level factors such as resource capacity of the health system, lack of accountability structures for intersectoral collaboration, and inadequate mechanisms for concurrent monitoring and evaluation of VHSNC were flagged as important determinants of VHSNC success. At the VHSNC level, member motivation to participate in meetings was flagged as important for well-functioning committees, as well as the influence of social hierarchies on participation of marginalized groups. One study emphasized that the key driver for improving motivation and commitment of members was the active engagement of the VHSNC president. Our study compares with these publications in two fundamental ways. First, in terms of methodology, our longitudinal research approach lends itself to studying VHSNC success in terms of processes of change rather than factors—an approach that draws on our earlier work on VHSNCs in Gumballi (Madon and Krishna 2017). Second, while the publications referred to in Table 3 focus on analysing VHSNC success at the level of implementation, they fall short of considering how implementation and policy mutually shape each other. For example, the vision and instrumentation of government in establishing the VHSNCs can be described as a process of policy formulation which has led to different degrees of enablement within these invited spaces as the basis for action. Together with this enablement is a parallel process of enactment that has unfolded at the field of action which we have characterized in terms of three key governance constructs of horizontal coordination, demand for accountability and self-help.

**Table 3.** Comparing recent VHSNC studies

Study	Methodology	VHSNC performance measures
Kumar *et al.* (2016)	Qualitative, cross-sectional, Uttar Pradesh	Active engagement of president; lack of motivation of members to participate actively; lack of operational guidelines’ intersectoral collaboration
Scott *et al.* (2017)	Qualitative, longitudinal over 1.5 years, rural North India	Social hierarchies at village level; resource and capacity deficits of health system; lack of operational capacity for intersectoral collaboration; fragmented lines of accountability
Ved *et al.* (2018)	Qualitative, based on a 2-year pilot experience, North and South India	State/district mechanisms needed for VHSNC capacity-building; for resource provision and fund release; capacity to monitor and learn from the field to provide feedback for policy
Dhiman *et al.* (2020)	Qualitative, cross-sectional, Himachal Pradesh	VHSNC member awareness about committee’s role; member responsibility; how funds were being used
Abdul Azeez *et al.* (2021)	Descriptive statistics, cross-sectional, Chattisgarh and Madhya Pradesh	Active participation of Gram Panchayat and members

Taken as a whole, our earlier research in Gumballi combines with the findings from the current study to build a complete theory of community health governance in which policy vision and instrumentation both shape and are shaped by the perceptions, tactics and practices of local actors interacting with each other. This theoretical contribution holds important implications for community health governance policy to which we now turn.

### Policy implications

Our study of community health governance as emergent phenomena can help to sensitize policymakers. Specifically, it can help promote dialogue about the government’s formulation of vision, the instruments it identifies and how these translate to field-level implementation. While the original intent behind the VHSNCs was to encourage community participation and collective action, our study provides greater insights about how participation happens, who acts and who reacts, and what are the issues that surface during the process. The VHSNCs were envisaged as a social space for intersectoral collaboration but in practice it is only when multiple stakeholders make sense of and feel motivated enough to exert agency for participating in discussions about village health that improvements in primary healthcare are possible. In the context of Gumballi, although we cannot make inferences about cause and effect, in the Appendix we see that there has been a continuous drop in diarrhoea since 2013 particularly after 2016 while average figures for Karnataka PHCs indicate no such systematic trend.

The findings from this study hold important policy implications for the promotion of intersectoral collaboration in primary healthcare as part of decentralization policy in developing countries. The village environment is an important place for learning about improving health and development and provides an opportunity for policymakers to assimilate the learning that accumulates from invited spaces which are established in order to make significant progress against debilitating water-borne diseases. Collective action by VHSNC members to improve intersectoral collaboration cannot be arrived at until a certain routine has been established, which requires a continuous process of training and capacity-building at VHSNC level to be institutionalized as these committees move from non-functional to well-functioning status. In all the five government documents we scrutinized, the capacity of government to monitor and learn from the field was only mentioned once calling for the need for greater policy focus on putting in place mechanisms to close the feedback loop from implementation back to policy. Current support for VHSNC capacity-building from the government seems highly inadequate in Gumballi as well as in our more recent research in Magadi taluk, Karnataka, where in the absence of capacity-building most of the VHSNCs still remain non-functional. A related point is the need for the government to re-evaluate how it measures VHSNC performance. While criteria such as membership frequency and attendance or the number of dysfunctional water pumps in the village provide a static snapshot of status, it is important to capture processual indicators that can help policymakers gain intelligence about the dynamics of change that are taking place at field level and how these interactions can help inform policy vision and instrumentation.

In terms of limitations, although the focus of our study was on integrative practices within invited spaces, we treated the 13 villages as a homogenous socio-political environment. For example, we did not compare and contrast the specific nuances of village politics and social hierarchies based on gender and caste within each village as this would constitute a separate endeavour. Our focus in this study was on the nature of interactions related to sanitation, nutrition and hygiene which we observed came up in all our study villages although the problem manifested itself in different ways as illustrated through our vignettes.

A second limitation relates to the scalability of our findings. We have engaged in intense, longitudinal fieldwork in which the sheer presence of our fieldworkers and their knowledge about the government’s intent and instrumentation with regard to the VHSNCs has no doubt influenced the building of capacity at village level. For example, our fieldworkers helped to establish VHSNCs in particular villages and tacitly motivate committee members and lay persons in all the study villages to participate in committee meetings. In contrast, what was observed at the field of action came from the interactions between VHSNC members themselves as they became sensitized about actions that could be taken to build capacity for improving village health. However, this approach is clearly not scalable as a model for capacity-building as while our study has been confined to 13 villages in Gumballi PHC in Karnataka alone there are 2193 PHCs covering approximately 32 895 villages. This raises the issue of the generalizability of the learnings about collective action and intersectoral collaboration from our study. The authors recognize this limitation and are currently working on devising a methodology whereby the lessons distilled from our research can be used for scaling up VHSNC capacity-building in other parts of Karnataka.

To conclude, our aim in this paper has been to improve theoretical understanding of community health governance structures as part of policy aimed at improving primary healthcare in LMICs. Conceived of in ministries housed thousands of miles away as the basis for action, these structures come alive at the field of action with possibilities to serve as powerful hubs for collective action and intersectoral collaboration. However, they deserve greater policy support to build capacity in order to sustain essential primary healthcare functions. Under conditions when daily practices are disrupted such as during the current Coronavirus 2019 (COVID-19) pandemic, the VHSNCs are today serving as a vital field of action for both community-based disease surveillance and containment during the pandemic, as well as for regular primary healthcare. We hope that our study will act as a catalyst for further process-oriented research in different country contexts to better understand how and why community health governance efforts become accepted as relevant forms of decentralized health governance.

## Supplementary Material

czac002_Supp

## Data Availability

The data underlying this article are available in the article and in its online supplementary material.

## Endnotes

^1.^
The NHM was launched in 2013 as a government programme that encompasses its two sub-missions, the National Rural Health Mission (NRHM) and the National Urban Health Mission.

^2.^
Kannada is the language spoken in Karnataka state.

^3.^
Gram Panchayat translates to village council in which members are elected to serve for a 5-year term.

^4.^
The NHM classifies marginalized members of the VHSNC in terms of those who have less voice such as women (health workers plus pregnant women and lactating mothers who are community members) and scheduled caste members.

^5.^
Anganwadi workers provide care for rural children.
