# Supplementary material for: Theorizing community health governance for strengthening primary healthcare in LMICs
Source: Health Policy Plan. 2022 Jan 25;37(6):706–16. doi: 10.1093/heapol/czac002 (PMC9189612; doi:10.1093/heapol/czac002)
Supplement: czac002_Supp [file czac002_supp.zip › HPP FINAL SUBMIT SUPPLEMENTARY.docx]

Appendix

Comparing the Incidence of Diarrhoea in Gumballi PHC with the rest of Karnataka

Source: Primary data for this graph was sourced from paper records kept at health outposts. For Karnataka PHC average, data was collected from Parliament records and from Directorate of Health Intelligence. We analysed the data using simple linear regression of annual disease data over time.
